# Metabolic Engineering Design Strategies for Increasing Acetyl-CoA Flux

**DOI:** 10.3390/metabo10040166

**Published:** 2020-04-23

**Authors:** Jason T. Ku, Arvin Y. Chen, Ethan I. Lan

**Affiliations:** 1 Institute of Molecular Medicine and Bioengineering, National Chiao Tung University, Hsinchu City 300, Taiwan; noeidting@gmail.com (J.T.K.); sssh10136@gmail.com (A.Y.C.); 2 Department of Biological Science and Technology, National Chiao Tung University, Hsinchu City 300, Taiwan

**Keywords:** acetyl-CoA, metabolic bypass, pyruvate dehydrogenase, coenzyme A

## Abstract

Acetyl-CoA is a key metabolite precursor for the biosynthesis of lipids, polyketides, isoprenoids, amino acids, and numerous other bioproducts which are used in various industries. Metabolic engineering efforts aim to increase carbon flux towards acetyl-CoA in order to achieve higher productivities of its downstream products. In this review, we summarize the strategies that have been implemented for increasing acetyl-CoA flux and concentration, and discuss their effects. Furthermore, recent works have developed synthetic acetyl-CoA biosynthesis routes that achieve higher stoichiometric yield of acetyl-CoA from glycolytic substrates.

## 1. Introduction

The rapid development of genetic and genomic tools allowed discoveries of the genes and enzymes associated with the production of industrially important chemicals. Methods and principles of metabolic engineering have then enabled the modification and transfer of the pathways that associate with these genes to almost any organism of choice to produce chemicals for industrial purposes. As these production pathways are optimized through both enzyme selection and engineering, the production is often limited by the availability of essential central metabolites. These essential metabolites are naturally produced from pathways optimized for cellular growth rather than for chemical production. As such, metabolic rewiring designs are necessary for increasing flux towards these essential metabolites.

Acetyl-Coenzyme A (acetyl-CoA) is a key two-carbon, ignoring the CoA moiety which only serves as an acyl-carrier, metabolite precursor for the production of lipids, polyketides, isoprenoids, amino acids, and numerous other bioproducts used in biochemical, biofuel, and pharmaceutical industries. At the same time, acetyl-CoA is also involved in numerous essential central metabolic pathways such as the TCA cycle, fatty acid biosynthesis, and amino acid biosynthesis. Pyruvate dehydrogenase (Pdh), the primary enzyme responsible for acetyl-CoA biosynthesis, is allosterically regulated with pyruvate [1] as an activator and acetyl-CoA [2] and reduced nicotinamide adenine dinucleotide (NADH) [3] as inhibitors. Therefore, intracellular flux and concentration of acetyl-CoA are highly regulated [4,5,6,7,8] to avoid potential metabolic burdens. Using the growth of *Escherichia coli* with glucose as an example, while flux towards acetyl-CoA has been measured to be about the same glucose uptake [9,10], this flux represents only about half of total glucose intake flux because of the stoichiometric ratio of glucose to acetyl-CoA is 1:2. In addition, its intracellular concentration is kept at relatively low levels. For example, intracellular acetyl-CoA in *E. coli* was reported with a concentration of 0.05–1.5 nmol/mg cell dry weight (CDW), corresponding to 20–600 μM [11]. This situation makes it difficult for the production pathways utilizing enzymes with high Km for acetyl-CoA. The acetyl-CoA concentration increases with the cell growth followed by gradually decreasing when reaching stationary phase [11]. In addition, acetyl-CoA concentration varies according to salt stress [12], temperature [12], pH [12], and oxygenic level [13]. Carbon source also affects intracellular acetyl-CoA concentration as it has been measured to be 0.82, 0.62, and 0.37 nmol/mg CDW, for growth on glucose, glycerol, and succinate, respectively [12]. Therefore, metabolic strategies for manipulating acetyl-CoA flux and concentration is highly desirable to deal with different cultivation conditions.

**Figure 1:**
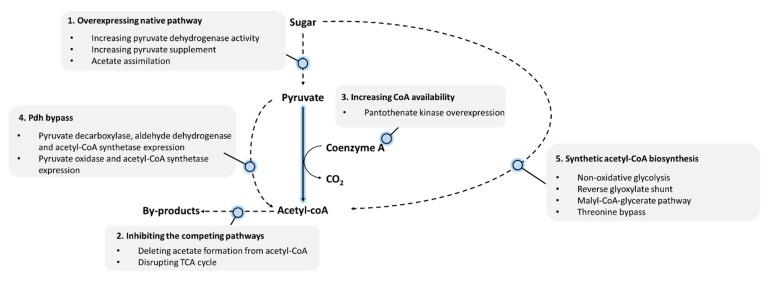
Summary of strategies for increasing acetyl-CoA flux and concentration in *Escherichia coli*. As acetyl-CoA is primarily synthesized from pyruvate, increase carbon flux toward pyruvate is an obvious strategy. Deletion of genes that are responsible for acetyl-CoA consumption is another commonly used strategy. Additionally, increase in CoA availability was demonstrated to functionally increase intracellular acetyl-CoA concentration. Since acetyl-CoA is primarily synthesized from pyruvate with an inevitable carbon loss, several pyruvate dehydrogenase bypasses were demonstrated to synthesize acetyl-CoA over theoretical yield. The bullet points listed in the gray area show the detail metabolic engineering method to meet each strategy. Dashed arrows indicate multi-step reactions.

To overcome the regulation of native acetyl-CoA biosynthesis, numerous strategies have been applied. This review summarizes the current approaches to increasing acetyl-CoA fluxes (Figure 1) from pyruvate and, in some cases, acetyl-CoA concentration as well. These approaches include overexpression of pyruvate dehydrogenase, increase of pyruvate supply, assimilation of acetate, inhibition of non-essential acetyl-CoA consumption, increase of CoA supply, and the construction of pyruvate dehydrogenase bypass. In addition, this review also discusses the recent development of novel metabolic pathways that lead to increased stoichiometric yield of acetyl-CoA from pyruvate through carbon conservation or additional carbon fixation. Together, the strategies presented in this work serves as a guide to metabolic engineering projects requiring acetyl-CoA as the metabolic precursor.

## 2. Overexpressing Native Pathways

Acetyl-CoA is primarily synthesized from pyruvate through pyruvate dehydrogenase (Pdh) aerobically with CO_2_ and NADH formation [14]. On the other hand, pyruvate formate lyase (Pfl) serves as the major enzyme for acetyl-CoA formation anaerobically with formate as byproducts in bacterial hosts such as *E. coli* [15]. Since pyruvate is the direct precursor for acetyl-CoA synthesis, the most straightforward strategy for increasing acetyl-CoA flux and concentration is to increase the activity of Pdh or Pfl. Alternatively, increasing carbon flux toward pyruvate also drives formation of acetyl-CoA.

### 2.1. Increasing Pyruvate Dehydrogenase Activity

**Figure 2:**
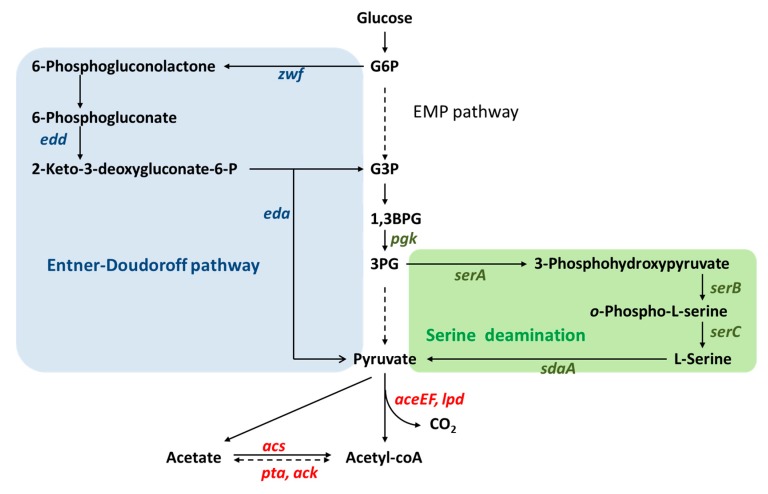
Pathways used to channel more carbon flux toward acetyl-CoA. Entner–Doudoroff pathway (ED pathway; blue shadowed area) is able to convert G6P into G3P and pyruvate independent from the conventional EMP pathway. Serine deamination (SD pathway; green shadowed area) on the other hand channeling pyruvate synthesis from the deamination of serine, which comes from the EMP pathway intermediate, 3PG. Overexpression of glycolysis, ED pathway, and SD pathway enhances pyruvate synthesis, which was expected to increase acetyl-CoA concentration since pyruvate is the precursor of acetyl-CoA. *aceEF* and *lpd* encode for pyruvate dehydrogenase, which is the primary enzyme responsible for acetyl-CoA synthesis. The italic labels represent the genes overexpressed for increasing carbon flux from sugar to acetyl-CoA. Dashed arrows indicate multi-step reactions. Abbreviations: *zwf*, glucose-6-phosphate dehydrogenase; *edd*, 6-phosphogluconate dehydratase; *eda*, 2-keto-3-deoxygluconate-6-phosphate aldolase; *pgk*, phosphoglycerate kinase; *serA*, phosphoglycerate dehydrogenase; *serB*, phosphoserine phosphatase; serC, phosphoserine; *sdaA,* L-serine deaminase; aminotransferase; *aceEF,* pyruvate dehydrogenase; *lpd,* lipoamide dehydrogenase (E3 subunit); *acs,* acetyl-CoA synthetase; *pta,* phosphate acetyltransferase; *ack,* acetyl-CoA kinase; G6P, glucose-6-phosphate; G3P, glyceraldehyde 3-phosphate; 1,3BPG, 1,3-bisphosphoglycerate; 3PG, 3-phosphoglycerate.

Pyruvate dehydrogenase complex is composed of three subunits, AceE, AceF, and Lpd (Figure 2). Although overexpression of Pdh complex has been thought to be difficult, its overexpression for increasing flux to acetyl-CoA has been applied to isoamyl acetate production in *E. coli*, which is a valuable ester that can be biosynthesized from the condensation of isoamyl alcohol and acetyl-CoA [16]. This study showed a 1.45-fold increase of the isoamyl acetate produced between Pdh overexpression strain and the parent strain, indicating the successful expression of Pdh. In a different study, the concentration of intracellular acetyl-CoA was measured with a 2-fold increase when Pdh was overexpressed [17]. In addition to Pdh overexpression, fine tuning its expression level through different copy number plasmids showed significant improvements in fatty acids production [18]. Pdh overexpression has also been achieved in model cyanobacterium *Synechococcus elongatus* PCC7942 with results of a 2-fold increase in acetyl-CoA concentration [19]. Contrary to the conventional notion that Pdh is difficult to overexpress, these studies have shown the positive effect of manipulating Pdh expression on intracellular acetyl-CoA flux and concentration.

In addition to genetic regulation, Pdh is competitively inhibited by NADH [20], which prohibits high activity under anaerobic condition even with overexpression. Therefore, Kim et al. constructed a Pdh mutant that is less sensitive to inhibition by NADH [21]. The NADH insensitive Pdh mutant was achieved by introducing a E354K mutation on the E3 subunit Lpd. A later study reported a 5-fold increase in carbon flux through Pdh using this NADH insensitive mutant under anaerobic condition when compared to wild-type [22]. Expression of this mutant Pdh has also aided the production of butanol by 1.6-fold [23]. Similarly, expression of a mutant Pdh homologue from *Klebsiella pneumonia* led to a 2-fold increase in 1,4-butanediol production under anaerobic conditions [24]. Altogether, these results showed that overexpression of Pdh increases flux toward acetyl-CoA, leading to higher production of acetyl-CoA derived compounds. The oxygen insensitive Pdh mutant further provides a promising tool for increasing acetyl-CoA anaerobically.

### 2.2. Increasing Pyruvate Flux Drives Downstream Acetyl-CoA Formation

Increasing carbon flux through glycolysis to pyruvate directly drives the downstream acetyl-CoA formation. To do so, enzymes of glycolysis are targets for genetic overexpression. Xu et al. [17] used OptForce, a Flux balance analysis procedure that directly uses the available flux measurements to predict necessary genetic manipulations for compound overproduction [25], and identified phosphoglycerate kinase (Pgk) and glyceraldehyde-3-phosphate dehydrogenase (GapA) as two glycolytic enzymes targets for overexpression to achieve increased flux towards pyruvate produced through glycolysis. Their data showed a near 30% increase in intracellular acetyl-CoA concentration upon overexpression of Pgk, leading to a near doubled flux towards their downstream product naringenin production [17].

In addition to glycolysis, typically referring to the Embden–Meyerhof–Parnas (EMP) pathway, other variants of glycolysis are also overexpression targets. For example, Zhang et al. [26] proposed a strategy of engineering the serine-deamination (SD) pathway and the Entner–Doudoroff (ED) pathway (Figure 2). Overexpression of *serABC* and *sdaA*, which enhances serine synthesis and serine deamination, improved pyruvate flux, leading to slight improvement on intracellular acetyl-CoA concentration by 10% [26]. Subsequently, a significant improvement on intracellular acetyl-CoA concentration was achieved by replacing the native promoter of the edd-eda operon, encoding for the ED pathway, with a constitutive promoter P_J23119_ and the native promoter of *zwf* with another constitutive promoter P_Trc-162_. Their resulting acetyl-CoA concentration therefore increased by 3-fold compared to the wild-type strain. Together with replacing native promoter of Pdh, their approach supported a relatively high flux production of poly-3-hydroxybutyrate (PHB), achieving a titer of 5.5 g/L [26]. Together, these studies showed the effect that enhancing glycolytic flux towards pyruvate has on increasing acetyl-CoA concentration and resulting flux of desired downstream products.

### 2.3. Acetate Assimilation into Acetyl-CoA

Acetate is a secreted metabolite under both aerobic [27] and anaerobic conditions [28] and represent a major acetyl-CoA flux loss. In model organisms such as *E. coli*, it is synthesized from either pyruvate through pyruvate oxidase P*oxB* or acetyl-CoA through phosphate acetyltransferase Pta and acetyl-CoA kinase Ack (Figure 2). To address this loss of acetyl-CoA flux, acetyl-CoA synthetase Acs, catalyzing ATP dependent CoA-acylation of acetate to acetyl-CoA, overexpression was proposed to assimilate the secreted acetate back to acetyl-CoA (Figure 2) [29]. Lin et al. [29] showed the overexpression of native *acs* in *E. coli* decreased the secreted acetate from 11 mM to a negligible level, indicating the efficient re-assimilation of acetate back to acetyl-CoA. Another study on fatty acid synthesis further showed that the intracellular acetyl-CoA concentration increased more than 3-fold, reaching 3.5 nmol/mg CDW, with *acs* overexpression when compared to the wild-type strain [30]. As an alternative, phosphate acetyltransferase (encoded by *pta*) and acetyl-CoA kinase (encoded by *ack*) were also used to assimilate acetate to acetyl-CoA [31,32] (Figure 2). By expressing the *pta-ack* operon, the N-acetylglutamate production was increased by 2-fold [31]. These studies show that both the expression of Acs or Pta and Ack are viable methods to re-assimilate acetate produced. In addition to assimilating secreted acetate, supplementation of acetate into culture medium makes *acs* expression more effective on increasing intracellular acetyl-CoA [30,33].

## 3. Inhibition of Competing Pathways

Inhibition of competing pathways is a conventional metabolic engineering design principle. In attempts to increase acetyl-CoA flux, it is also a core design strategy used. However, pathways for the biosynthesis of essential metabolites such as fatty acids and amino acids using acetyl-CoA cannot be knocked out while maintaining suitable cellular growth rate. Although the rapid development of genetic tools such as sRNA [34] and CRISPRi [35] may allow transient and controlled partial inhibition, precise control is still challenging. As a result, most studies focus on inhibiting non-essential, but obvious and with large flux, pathways.

### 3.1. Deletion of Phosphate Acetyltransferase and Acetyl-CoA Kinase to Reduce Acetate Production

**Figure 3:**
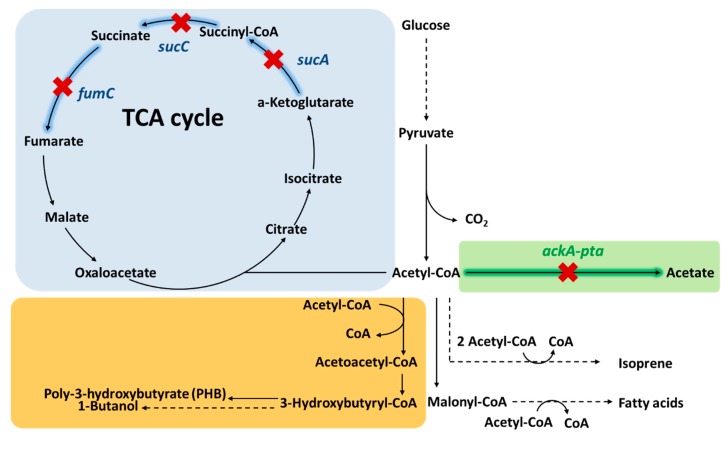
Genes inhibited to decrease acetyl-CoA consumption. Since acetate is a byproduct synthesized by *E. coli* under both aerobic and anaerobic condition, the acetate formation genes, *ackA* and *pta*, were frequently deleted to avoid the consumption of acetyl-CoA to acetate. *ack* and *pta* encode the enzyme of acetyl-CoA kinase and phosphate acetyltransferase, respectively. In addition, acetyl-CoA is consumed significantly by TCA cycle aerobically. Therefore, the deletion of *sucA*, encoding for α-ketoglutarate dehydrogenase, *sucC*, encoding for succinyl-CoA synthetase and *fumC*, encoding for fumarase, were also used for increasing acetyl-CoA concentration. The orange shadowed area represents the CoA-dependent pathway for 1-butanol and poly-3-hydroxybutyrate (PHB) synthesis, whose production improved by applying the deletion of acetyl-CoA consuming pathways. Dashed arrows indicate multi-step reactions.

To block the acetate formation from acetyl-CoA, *ackA-pta* was the most frequently disrupted operon (Figure 3). Vadali et al. showed that the deletion of *ackA-pta* operon increased intracellular acetyl-CoA concentration by 16% with a residual acetate secretion of 4.5% compared to that of parent strain under anaerobic condition [36]. In conjunction with expression of an acetyl alcohol acetyltransferase (ATF2) and the supplementation of isoamyl alcohol, this strategy effectively increased isoamyl acetate, requiring acetyl-CoA as a direct precursor, production by 4.8-fold compared to the parent strain [36].

In addition to pathways using acetyl-CoA as direct precursor to the desired product such as isoamyl acetate, other products requiring acetyl-CoA as a further upstream metabolite also benefit from *ackA-pta* operon knockout. One such pathway is the CoA-dependent chain extension pathway (Figure 3). Since both poly-hydroxybutyrate (PHB) and 1-butanol are both synthesized from two molecules of acetyl-CoA via CoA-dependent carbon chain extension (Figure 3), acetyl-CoA availability is believed to be an important parameter for high PHB [37] or 1-butanol synthesis [38]. Although the exact acetyl-CoA concentration and flux were not measured in the study of PHB biosynthesis, deletion of *ack-pta* increased the PHB production yield by 48% with the decreased of acetate formation [39]. 1-butanol production, on the other hand, was reported a significant improvement on titer by 10-fold with *pta* deletion [40]. A detailed metabolomics analysis was performed to the 1-butnaol production [41], showing that *pta* deletion increased intracellular acetyl-CoA concentration by 2-fold. In addition, this same study also measured the concentrations of other CoA intermediates in the CoA-dependent butanol synthesis pathway. Their results revealed that the CoA regeneration was inefficient enough due to the low activity of butyryl-CoA reductase, which probably limited the acetyl-CoA flux. After addressing the CoA recycling issue by expressing a higher activity reductase, the resulting strain showed a further 2-fold increase in intracellular acetyl-CoA concentration along with a 2-fold improvement in butanol production compared to the strain using original butyryl-CoA reductase [41]. These studies showed the effectiveness of *pta-ack* knockout for streamlining carbon flux to acetyl-CoA and its downstream products. Furthermore, the metabolomics study on butanol production also suggested the importance of CoA recycling in order to keep its availability for acetyl-CoA turnover from pyruvate, leading to a higher acetyl-CoA flux.

Deletion of acetate formation was also applied to fatty acid [42] and isoprene production [43] (Figure 3). Since fatty acid biosynthesis requires acetyl-CoA and malonyl-CoA, which is also derived from acetyl-CoA, as precursors, deletion of *pta* itself increased total fatty acid synthesis titer by 1.5-fold [44] (Figure 3). In the case of isoprene production, three acetyl-CoA molecules are required using the mevalonate pathway. Kim et al. [43] showed initial demonstration of isoprene production using *E. coli* resulted in 7.4 g/L of acetate produced as by-product. Upon deletion of the *pta-ack* operon, acetate formation was reduced to 1.4 g/L while increasing isoprene production by about 2-fold [43]. A similar strategy of deleting phosphate acetyltransferase has been also applied to other chemical productions, such as acetaldehyde [45], itaconate [46], poly-3-hydroxybutyrate (PHB) [39,47,48], butyraldehyde [49], acetone [50], and butanone [50] biosynthesis. This approach is one of the most well-demonstrated methods for enhancing acetyl-CoA supply.

### 3.2. Disrupting TCA Cycle

The TCA cycle represents a large acetyl-CoA drain especially during aerobic metabolism. It can be disrupted by inhibiting enzymes downstream of α-ketoglutarate, particularly α-ketoglutarate dehydrogenase. Inhibition of the α-ketoglutarate dehydrogenase was achieved through deletion of *sucA* gene which codes for the E1 subunit. This strategy was used to improve the yield of mevalonate production [51]. In the same Xu et al. study [17] mentioned previously in Section 2.2 that used Optforce [25] to optimize acetyl-CoA flux, succinyl-CoA synthetase and fumarase were identified as two targets needed to be knocked down. Through deletion of *sucC* and *fumC*, coding for succinyl-CoA synthetase and fumarase, respectively, acetyl-CoA concentration increased by more than 2-fold compared to the strain without these deletions. Together with overexpression of Pdh, increasing pyruvate flux, and acetyl-CoA carboxylase expression, their product naringenin titer improved from 85.5 to 474 mg/L, demonstrating the beneficial effect of increased acetyl-CoA availability on polyketide synthesis. Genes coding for TCA cycle enzymes responsible for the synthesis of α-ketoglutarate are generally not deleted due to inability to grow under defined media by the resulting stain. Reduced carbon flux to α-ketoglutarate also impaired cellular growth [52]. Nonetheless, citrate synthase knockout has been shown to increase acetyl-CoA availability for citramalate synthesis, which requires acetyl-CoA as the direct precursor [53]. The downside such approach is the required supplement of glutamate in culture medium. Together, these studies showed the versatility of deleting the competing pathway to increase acetyl-CoA concentration and flux.

## 4. Increasing CoA Availability

**Figure 4:**
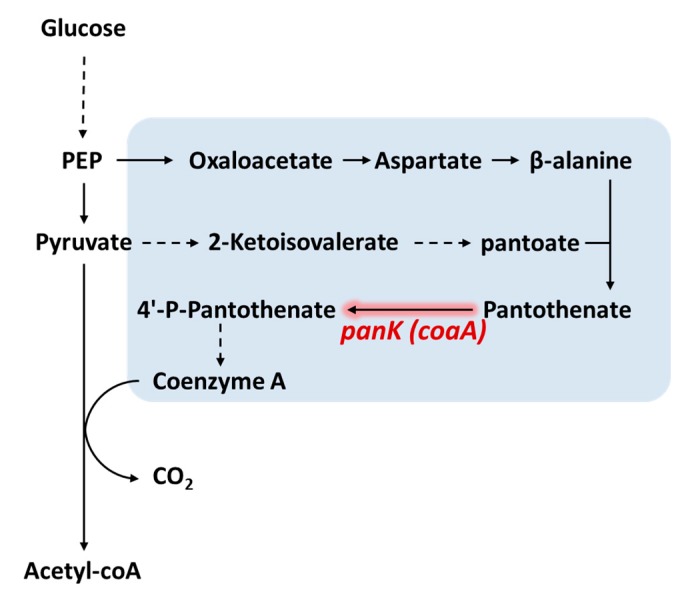
Scheme of coenzyme A synthesis. The coenzyme A synthesis pathway is shadowed in blue. *panK* (also named as *coaA*) was the committed step for coenzyme synthesis, which is proposed to be expressed to increase both coenzyme A and acetyl-CoA concentration. Dashed arrows indicate multi-step reactions. Abbreviation: PEP, phosphoenolpyruvate.

Concentration of intracellular CoA is the upper limit to that of acetyl-CoA. Natural intracellular acetyl-CoA concentration may be insufficient to drive high flux conversion to its downstream products in some cases due to higher Km of the enzymes involved. For example, the Km of CoA-acylating aldehyde dehydrogenase used in ethanol production from *Salmonella enterica* is 342 μM [54], which is around or higher than the natural acetyl-CoA concentration in model hosts such as *E. coli*. To override the native regulation [55] that controls intracellular CoA concentration, Vadali et al. [56] proposed to increase intracellular acetyl-CoA and CoA concentration by overexpressing the native *panK* gene to avoid the regulation in *E. coli*. Pantothenate kinase (PanK; also named as CoaA) is the most regulated step in CoA synthesis [57,58] and is sensitively feedback inhibited by CoA pool [59,60] (Figure 4). The acetyl-CoA concentration in the *panK* overexpression strain increased by 3-fold compared to the control strain [56]. This strategy was further applied to isoamyl acetate production [61]. By combining the competing pathway knockout with increasing CoA availability, isoamyl alcohol production was increased by 2.27-fold compared to the control strain without any acetyl-CoA concentration elevating strategy. Notably, the intracellular CoA and acetyl-CoA concentration increased by 3.17-fold and 2.27-fold in the engineered strain, respectively.

Expressing pantothenate kinase to increase intracellular acetyl-CoA for improving bioproduction was also applied to succinate biosynthesis [62]. Since succinate is an intermediate of the TCA cycle, whose substrate is acetyl-CoA and oxaloacetate, increased acetyl-CoA concentration was expected to improve succinate production. Furthermore, PEP carboxylate (Ppc) and pyruvate carboxylase (Pyc), enzymes responsible for oxaloacetate synthesis and key to succinate production, were reported to require acetyl-CoA for activation [63,64]. Therefore, higher acetyl-CoA concentration aided in the activity of Ppc and Pyc. *panK* and *ppc* overexpression led to increased acetyl-CoA concentration from 0.24 to 8.05 μM and accompanied by a 30% improvement in succinate production [62].

Recently, the CoA and acetyl-CoA contents in *E. coli* strain expressing pantothenate kinases from different organisms, including *Staphylococcus aureus* (*Sa_coaA*), *Pseudomonas putida* (*Pp_coaA*), and *E. coli* (*Ec_coaA*), were further investigated [65]. The results showed that the intracellular acetyl-CoA content can be efficiently enhanced by expressing any of the *coaA* genes. While the expression of *Sa_coaA* resulted in a similar amount of acetyl-CoA to the strain that expressed *Ec_coaA*, the expression of *Pp_coaA* led to the highest amount of acetyl-CoA and total CoA content, achieving about 3-fold compared to the strain without pantothenate kinases overexpression under the same condition. Notably, acetyl-CoA occupied more than 70% of the total CoA content whether the pantothenate kinases were overexpressed or not, indicating the feasibility of increasing CoA availability to improve acetyl-CoA derived biochemical production. Although most of the studies expressed pantothenate kinase from *E. coli* to increase acetyl-CoA, this study showed the pantothenate kinase from *P. putida* may be a better candidate.

## 5. Construction of Pyruvate Dehydrogenase Bypass

**Figure 5:**
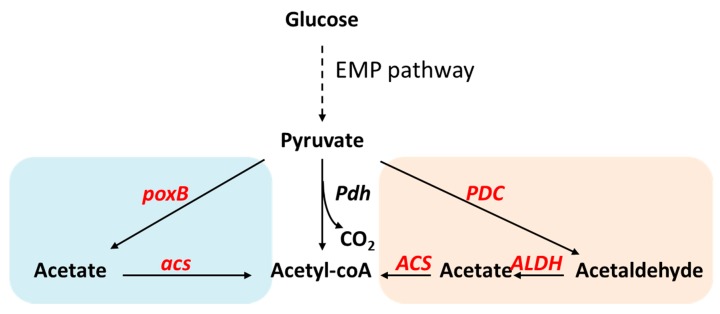
Scheme of pyruvate dehydrogenase (Pdh) bypass. Pdh bypass allows acetyl-CoA synthesis from pyruvate independent from Pdh. The pink area was naturally occuring in *Saccharomyces cerevisiae*, providing acetyl-CoA in cytosol. The blue shadowed area, on the other hand, was first used in *E. coli*. Both of the Pdh bypass versions were used to increase acetyl-CoA concentration for metabolic engineered bioproduction. Abbreviation: *poxB,* pyruvate oxidase; *acs,* acetyl-CoA synthetase; *PDC*, pyruvate decarboxylase; *ALDH,* aldehyde dehydrogenase.

Although several studies have shown the positive effect of pyruvate dehydrogenase overexpression, Pdh is nonetheless regulated by a variety of allosteric effectors [3], which may be difficult to completely control. Therefore, as an alternative, a bypass pathway consisting of pyruvate decarboxylase, acetaldehyde dehydrogenase, and acetyl-CoA synthetase (Figure 5) was used to convert pyruvate to acetyl-CoA. This pathway was particularly useful for the metabolic engineering of yeasts in productions that require acetyl-CoA [66]. In model yeasts such as *Saccharomyces cerevisiae,* Pdh is located in mitochondria whereas most biosynthesis occurs in the cytosol. Therefore, through the construction of this Pdh-bypass, acetyl-CoA concentration was increased from around 0.36 nmol/g CDW to more than 1.1 nmol/g CDW, representing a 3-fold improvement [67]. The expression of this bypass was shown to improve mevalonate production from 1.78 to 2.52 mM [66]. A different Pdh bypass was constructed in *E. coli* through the expression of pyruvate oxidase PoxB and acetyl-CoA synthetase Acs. Using the Pdh bypass, acetyl-CoA is synthesized from acetate, which comes from the decarboxylative oxidation of pyruvate (Figure 5). This version of the Pdh bypass was shown to improve intracellular acetyl-CoA concentration by 2-fold [68], which led to an increase of isopropanol production.

Photoautotrophic hosts such as cyanobacteria usually do not possess obvious acetyl-CoA drains such as the *pta-ack* operon. As a result, pathways requiring large acetyl-CoA flux such as the CoA-dependent chain extension pathway for butanol and butyrate production is limited and requires alternative pathway engineering [69,70]. Nonetheless, Pdh bypass has recently been engineered into model cyanobacterium *Synechococcus elongatus* PCC 7942 and helped to increase the intracellular acetyl-CoA concentration from 17 to 138 µg/g CDW [71]. Together, Pdh bypass increases intracellular acetyl-CoA by serving as an alternative for acetyl-CoA synthesis from pyruvate with same carbon yield. However, depending on the pathway for acetyl-CoA derived compound synthesis, the ATP consumption during the activation of acetate to acetyl-CoA in Pdh bypass may lower the production yield.

## 6. Synthetic Acetyl-CoA Biosynthesis

**Figure 6:**
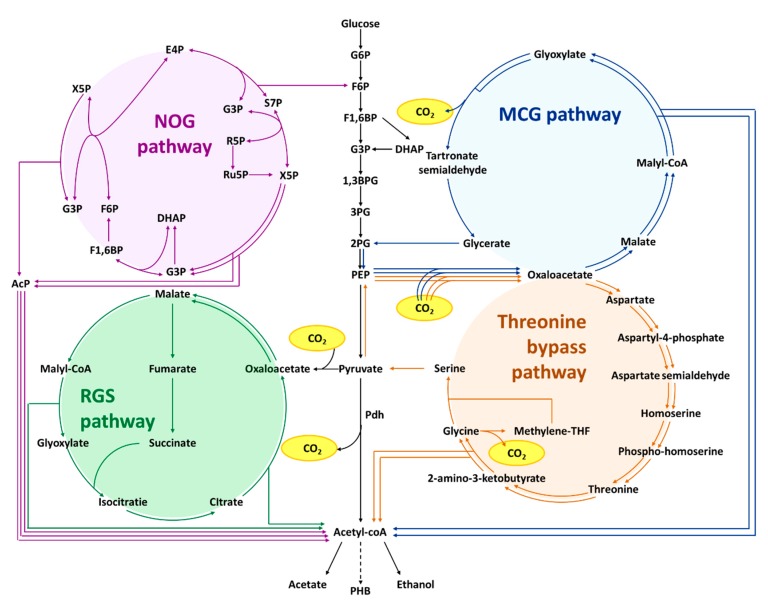
Synthetic Pdh bypass to increase the acetyl-CoA yield from sugar. The purple arrows indicate the non-oxidative glycolysis (NOG) pathway, which converts sugar phosphate to the acetyl-phosphate or further acetyl-CoA in a stoichiometry amount. The green arrows indicate the reverse glyoxylate shunt (rGS), leading to two acetyl-CoA from one pyruvate. The blue arrows indicate the malyl-CoA-glycerate (MCG) pathway, which net converts one phosphoenolpyruvate (PEP) to two acetyl-CoA. The orange arrows indicate the threonine bypass, which is able to convert one PEP into two acetyl-CoA. Redox equivalents and energy requirements of each reaction are not shown in this scheme. Abbreviations: G6P, glucose-6-phosphate; F6P, fructose-6-phosphate; F1,6P, fructose-1,6-bisphosphate; DHAP, dihydroxyacetone phosphate; G3P, glyceraldehyde 3-phosphate; 1,3BPG, 1,3-bisphosphoglycerate; 3PG, 3-phosphoglycerate; 2PG, 2-phosphoglycerate; PEP, phosphoenolpyruvate; X5P, xylulose-5-phosphate; E4P, erythrose-4-phosphate; S7P, sedoheptulose-7-phosphate; R5P, ribose-5-phosphate; Ru5P, ribulose-5-phosphate.

Although various strategies described above successfully increased acetyl-CoA concentration or flux, conversion of pyruvate to acetyl-CoA is coupled to an inevitable loss of carbon. Due to the decarboxylation of pyruvate, the stoichiometric yield of acetyl-CoA from glucose is 2 mole per mole of glucose. To go beyond this stoichiometric yield, novel synthetic pathways (Figure 6) are constructed to conserve or fix additional carbon into acetyl-CoA.

### 6.1. Non-Oxidative Glycolysis (NOG)

The non-oxidative glycolysis [72] is a synthetic pathway that completely conserves carbon when converting glucose to acetyl-CoA. It allows for the production of three acetyl-CoA molecules from a glucose. The key enzyme involved is a phosphoketolase which can cleave either fructose-6-phosphate (F6P) or xylulose-5-phosphate (X5P) into erythrose-4-phophate (E4P) or glyceraldehyde-3-phosphate (G3P), respectively, and acetyl-phosphate [73]. Represented in Figure 6 is the version of the non-oxidative glycolysis (NOG) pathway based on X5P cleavage. Acetyl-phosphate is then converted into acetyl-CoA through phosphate acetyltransferase Pta. Through sugar-phosphate rearrangement naturally used in both the pentose phosphate pathway or the Calvin cycle (Figure 6), a net reaction of one F6P to three acetyl-phosphate is possible. Furthermore, the phosphoketolase catalyzed reaction is generally irreversible and therefore provided a driving force for this pathway. Using this pathway, Bogorad et al. [72] showed a production of 3.6 g/L acetate from xylose with a yield of 2.2 (mole/mole xylose), which was above the theoretical yield of 1.67 (mole/mole xylose) using native glycolysis. Notably, although the intracellular acetyl-CoA concentration was not quantified, the NOG strain was reported with an intracellular acetyl-phosphate concentration of 10 mM, which can be easily converted to acetyl-CoA. On the other hand, the strain without NOG pathway expression only has acetyl-phosphate concentration of below 1 mM, showing the efficient acetyl-phosphate generation by the NOG pathway. Recently, by combining rational design, genome editing, and evolution, the same group was able to implement this NOG pathway into *E. coli* for EMP glycolysis independent growth [74]. The resulting strain, containing 11 gene overexpressions, 10 gene deletions by design, and more than 50 genomic mutations (including three global regulators), was able to convert 83% carbon from glucose into acetate [74]. While complete carbon conservation implies no reducing cofactors can be produced, this pathway offers a great potential to significantly increase the yield of acetyl-CoA derived product with the addition of an external electron source such as hydrogen.

### 6.2. Reverse Glyoxylate Shunt (rGS)

In addition to carbon conservation pathway, a synthetic reversed glyoxylate shunt [75] was also engineered to convert one pyruvate molecule into two acetyl-CoA, thereby achieving carbon fixation (Figure 6). Pyruvate is first carboxylated into a four-carbon oxaloacetate, that is subsequently reduced and cleaved into two acetyl-CoA molecules. To most model prokaryotes, this pathway requires introduction of only two enzymes: malate thiokinase and malyl-CoA lyase. This pathway has been demonstrated through conditional auxotrophies of TCA intermediates. An oxaloacetate/aspartate auxotrophic *E. coli* was rescued by this pathway using malate and succinate [75], indicating the functional expression of the pathway. While this pathway shows promise for substantially increasing stoichiometric yield of glucose to acetyl-CoA to 1:4 with addition of an external electron source such as hydrogen due to two acetyl-CoA produced from one pyruvate, it is still in early stages of development and has yet to be characterized in more detail.

### 6.3. Malyl-CoA-Glycerate Pathway (MCG Pathway)

The Malyl-CoA glycerate (MCG) pathway [76] achieved a similar effect as that of the reverse glyoxylate shunt (rGS) pathway mentioned above. This synthetic pathway starts from carboxylating two phosphoenolpyruvate (PEP) to two oxaloacetate molecules. Subsequently, the two oxaloacetate is reduced and activated into malyl-CoA, similar to that of rGS. Malyl-CoA is cleaved into acetyl-CoA and glyoxylate. Here, two glyoxylates are then used to regenerate PEP through tartronate semialdehyde (Figure 6). The overall reaction using MCG pathway produces two molecule acetyl-CoA from a molecule PEP with three molecule NADH and two molecule ATP consumption. Similar to the rGS, using this pathway would increase the stoichiometric yield of glucose to acetyl-CoA from 1:2 to 1:4 with addition of external electron source such as hydrogen. This pathway is easier to construct than the rGS because it does not contain a bifurcation at the malate node that the rGS has. With the expression of MCG pathway, Yu et al. showed that *E. coli* was able to produce acetate and ethanol with yield of 2.9 (mole C2 compound/mole glucose), which is over the theoretical yield using the native acetyl-CoA synthesis pathway [76]. This functional demonstration of exceeding natural stoichiometric yield outperforms the rGS.

### 6.4. Threonine Bypass

Lin et al. [77] constructed a threonine bypass based on flux balance analysis (FBA) to increase the intracellular acetyl-CoA flux for Poly-hydroxybutyrate production (Figure 6). The net effect of this pathway is identical to the rGS and MCG pathways where one mole of PEP converts to two moles of acetyl-CoA. The pathway started from carboxylating phosphoenolpyruvate (PEP) into oxaloacetate followed by a threonine synthesis pathway. Then, the threonine is oxidized to 2-amino-3-ketobutyrate followed by the cleavage into acetyl-CoA and glycine. The glycine is then converted to serine via a THF mediated reaction. Finally, serine was deaminated into pyruvate, which can be used to regenerate PEP. In their work, Lin et al. [77] showed a 3-fold increase in intracellular acetyl-CoA concentration upon the expression of this threonine bypass, resulting in a Poly-hydroxybutyrate titer increase from 2.08 to 5.97 g/L.

Together with the above-mentioned rGS and MCG pathways, these three pathways are capable of producing two molecules of acetyl-CoA from one molecule of PEP or pyruvate. If supplemented with inorganic electron source such as hydrogen, the maximum stoichiometric yield from glucose becomes 1:4. While functional demonstration and applications of these pathways in producing acetyl-CoA based compounds are still in its early developmental stages, these pathways offer substantial promise. Currently, these pathways require additional modifications such as identification and increase the rate limiting step in order for them to be more widely applied. Furthermore, as with the development of the NOG pathway [72,74], optimized implementation of these pathways may require a combination of rational design, genome editing, and evolution, to truly enable high performance of acetyl-CoA derived compound productions.

## 7. Conclusions

Recent advances on metabolic engineering provides the production of wide variety of compounds. As an essential central metabolite, acetyl-CoA serves as the precursor to various chemicals while supporting cellular growth. In the past decades, significant efforts have focused on increasing flux and concentration of intracellular acetyl-CoA in order to support microbial chemical synthesis. Conventionally, overexpressing the native pathway for acetyl-CoA synthesis and deletion of competing pathways are the most commonly used strategies. As an alternative, providing CoA for efficient acetyl-CoA synthesis was also demonstrated to function on increasing acetyl-CoA flux.

Recently, advances in bioinformatics and metabolic engineering tools enables further bold design of synthetic pathways, such as the NOG pathway, rGS, MCG pathway, and threonine bypass, allowing over theoretical yield of acetyl-CoA synthesis from sugar. While these synthetic pathways have been demonstrated in their respective studies, their optimized integration into microbial cell factories still require additional work.

Acetyl-CoA flux and concentration remains a tricky challenge to metabolic engineers. As a central primary metabolite, its complex interaction with transcriptome and proteome has yet to be completely deciphered. For example, a recent study on threonine synthesis showed that acetyl-CoA concentration increased significantly by 2.1-fold with the expression of the PHB synthetic pathway [78]. Although the increased acetyl-CoA concentration can be explained by downregulation of *pta-ack* operon and upregulation of *acs*, its molecular basis remains unclear.

Although this review primarily discusses studies using *E. coli* as an example to show the strategies for increasing acetyl-CoA flux and concentrations, these strategies can also be applied to other organisms with appropriate modification. For example, eukaryotes, such as *S. cerevisiae,* synthesize acetyl-CoA in mitochondria without the ability of transporting to cytosol, Pdh bypass [66], or ATP citrate lyase mediated acetyl-CoA synthesis [79] hence was required to increase cytosol acetyl-CoA content. Pdh overexpression was used in *S. serevisiae* to increase acetyl-CoA concentration [80], however, required lipoic acid supplementation.

In conclusion, the effects of the approaches discussed in this review and summarized in Table 1 are convincing as these works either directly quantified intracellular acetyl-CoA concentration or showed a significant increase of their acetyl-CoA derived bioproducts (Table 2).

## Figures and Tables

**Table 1:** Strategies for increasing acetyl-CoA and acetyl-CoA based products.

Approach	Strategy	Genetic Manipulations	Rational	Example
Overexpressing native pathways	Increase pyruvate dehydrogenase activity	Overexpressing *aceEF*, *lpd*	Overexpression of pyruvate dehydrogenase to increase activity	[16,17]
		Lpd * (E354K)	Introducing the mutation in E3 subunit of Pdh increased anaerobic activity due to insensitive to NADH	[22,23]
	Increase pyruvate supplement	△*serC*::P*_trc-162_*::*pgk*, *serABC*;	Entner–Doudoroff pathway converts glucose-6-phopshate to pyruvate using the genes, *zwf*, *edd*, and *eda*	[26]
		P*_sdaA_*::P*_trc_*; P*_edd-eda_*::P_J23119_; P*_zwf_*::P*_trc-162_*;	Pgk helps to provide 3PG from 1,3BPG. Then, the serine-deamination pathway converts 3PG into pyruvate using the genes *serABC* and *sdaA*.	[26]
	Assimilate acetate	Overexpressing *acs* or *pta-ack*	Activating acetate into acetyl-CoA, allowing the assimilation of naturally secreted or supplemented acetate	[31,32,33]
Inhibiting the competing pathway	Deleting acetate formation from acetyl-CoA	△*pta*	Deletion of phospho acetyltransferase blocks the consumption of acetyl-CoA into acetate	[40,42,43]
	Disrupting TCA cycle	△*sucA*	Deletion of α-ketoglutarate dehydrogenase (*sucA*) disrupts the TCA cycle but maintains glutamate synthesis	[51]
		△*scuC* △*fumC*	Deletion of succinyl-CoA synthetase (*sucC*) and fumarase (*fumC*) disrupt the TCA cycle	[17]
		△*gltA*	Deletion of citrate synthase to block the first step of the TCA cycle	[53]
Enhancing CoA availability	Enhance CoA synthesis	Overexpressing *panK*	Pantothenate kinase serves as the limiting step of CoA synthesis	[56,61]
Pdh bypass	Pdh bypass	Overexpressing *PDC*, *ACDH*, and *ACS*	PDC, ACDH, and ACS allows the conversion of pyruvate to acetyl-CoA via acetaldehyde and acetate as intermediate	[66]
		Overexpressing *poxB* and *acs*	poxB and acs allow the conversion of pyruvate to acetyl-CoA via acetate as intermediate	[68]
Synthetic acetyl-CoA biosynthesis	Non-oxidative glycolysis	Δ*adhE*, Δ*ldhA*, Δ*frdBC*, Δ*pflB*, P_LlacO1_::*fbp*, *fxpk*	Expression of fructose 1,6-bisphosphatase and phosphoketolase, allowing the conversion of glucose-phosphate to stoichiometry amount acetyl-phosphate or acetyl-CoA	[72]
	Non-oxidative glycolysis	Evolved strain with 50 genomic mutation and P_LlacO1_::*xpk_BA_* *, *glf_ZM_*, *glk*, *tkt2_MB_*, *tal_KP_*, *glpX* P_LlacO1_::*tkt2_MB_*, *tkt1_MB_*	Combining evolution, the NOG pathway supports *E. coli* growth and nearly complete carbon conservation from glucose to acetyl-phosphate or acetyl-CoA	[74]
	Reverse glyoxylate shunt	ΔgltA Δmdh Δppc ΔcitE Δmqo ΔaceB Δicd P_LlacO1_::DctA, PLlacO1::AceA; P_LlacO1_:AcnA; P_LlacO1:_:AclB, AclA; P_LlacO1:_:SucCD-2, Mcl1	rGS provides a net conversion of C4 compound such as malate into two acetyl-CoA	[75]
	Malyl-CoA-glycerate cycle	∆*aceB* ∆*glcB* ∆*frdB* ∆*ldhA* ∆*pstG* P_LlacO1_::*mtkB*, *mtkA(M.c)*, *mcl*; P_LlacO1_::*gcl*, *hyi* P_LlacO1_::*garK*, *mdh* P_LlacO1_::*ppc*	PEP carboxylase, malate dehydrogenase, malate thiokinase, and malyl-CoA lyase together with glyoxylate assimilation pathway allows two acetyl-CoA from PEP	[76]
	Threonine bypass	P*_kbl-tdh_*::P*_trc_*, P*_sdaA_*::P*_trc_*, *thrA*::*thrA* * (C1034T), P*_thrABC_*::P_Trc-162_, P*_ppc_*::P*_tac_*, P*_glyA_*::P_Trc-162_	Expression of threonine synthesis pathway and threonine degradation converts PEP to acetyl-CoA and glycine, which is next converted to pyruvate. Together, generating an extra acetyl-CoA.	[77]

* Mutant enzyme.

**Table 2:** The effects of applying acetyl-CoA increasing strategy on biochemical production.

Acetyl-CoA Derived Biochemical	Acetyl-CoA Increasing Strategy Applied	Fold Increased	Titer Achieved	Reference
Isoamyl acetate	Increasing pyruvate dehydrogenase activity; gene deletion to reduce acetate formation	1.5	0.023 g/L	[16]
	Gene deletion to reduce acetate formation	4.8	0.23 g/L	[36]
	Gene deletion to reduce acetate formation; increasing CoA availability	2.3	0.144 g/L	[61]
Isopropanol	Pyruvate dehydrogenase bypass	4.4	3.8 g/L	[68]
1,4-Butanediol	Increasing pyruvate dehydrogenase activity	2.0	7.5 g/L	[24]
1-Butanol	Increasing pyruvate dehydrogenase activity	1.6	4.3 g/L	[23]
	Gene deletion to reduce acetate formation	10	30 g/L	[40]
Poly-3-hydroxybutyrate	Increasing pyruvate flux; increasing pyruvate dehydrogenase activity	2.7	5.5 g/L	[26]
	Threonine bypass	2.9	5.97 g/L	[77]
N-Acetylglutamate	Gene deletion to reduce acetate formation; acetate assimilation	2.0	17.8 g/L	[31]
3-Hydroxypropionate	Acetate assimilation	2.5	0.25 g/L	[33]
Citramalate	Gene deletion to reduce acetate formation; gene deletion to disrupt TCA cycle	1.2	19.8 g/L	[53]
Succinate	Increasing CoA availability	1.3	2.7 g/L	[62]
Fatty acid	Increasing pyruvate flux; increasing pyruvate dehydrogenase activity; gene deletion to disrupt TCA cycle	5.6	0.47 g/L	[17]
	Acetate assimilation	2.2	0.68 g/L	[30]
Mevalonate	Pyruvate dehydrogenase bypass	1.4	0.37 g/L	[66]
Isoprene	Gene deletion to reduce acetate formation	2.0	1.8 g/L	[43]
